# Kinetic and Thermodynamic Aspects of the Degradation of Ferritic Steels Immersed in Solar Salt

**DOI:** 10.3390/ma17235776

**Published:** 2024-11-25

**Authors:** Rafael Felix-Contreras, Jonathan de la Vega Olivas, Cinthya Dinorah Arrieta-Gonzalez, Jose Guadalupe Chacon-Nava, Roberto Ademar Rodriguez-Diaz, Jose Gonzalo Gonzalez-Rodriguez, Jesus Porcayo-Calderon

**Affiliations:** 1 Departamento de Ingenieria Quimica y Metalurgia, Universidad de Sonora, Hermosillo 83000, Sonora, Mexico; a223230118@unison.mx (R.F.-C.); jonathan.delavega@unison.mx (J.d.l.V.O.); 2 Tecnológico Nacional de Mexico/Instituto Tecnológico de Zacatepec, Calzada Instituto Tecnológico 27, Zacatepec 62780, Morelos, Mexico; cinthya.ag@zacatepec.tecnm.mx; 3 Centro de Investigación en Materiales Avanzados (CIMAV), Miguel de Cervantes 120, Complejo Industrial Chihuahua, Chihuahua 31136, Chihuahua, Mexico; jose.chacon@cimav.edu.mx; 4 Tecnologico Nacional de Mexico/Tecnologico de Estudios Superiores de Coacalco, Av. 16 de Septiembre 54, Col. Cabecera Municipal, Coacalco de Berriozábal 55700, Estado de Mexico, Mexico; ademar@tesco.edu.mx; 5 Universidad Autonoma del Estado de Morelos-CIICAp, Av. Universidad 1001, Col. Chamilpa, Cuernavaca 62209, Morelos, Mexico; ggonzalez@uaem.mx

**Keywords:** ferritic steels, solar salt, corrosion, thermodynamic, activation energy, polarization curves

## Abstract

The study and improvement of the corrosion resistance of materials used in concentrated solar power plants is a permanent field of research. This involves determining their chemical stability when in contact with heat transfer fluids, such as molten nitrate salts. Various studies indicate an improvement in the corrosion resistance of iron-based alloys with the incorporation of elements that show high reactivity and solubility in molten nitrate salts, such as Cr and Mo. This study analyzes the kinetic and thermodynamic aspects of the beginning of the corrosion process of ferritic steels immersed in Solar Salt at 400, 500, and 600 °C. The analysis of the kinetic data using the Arrhenius equation and the Transition State Theory shows that an increase in the Cr/Mo ratio reduces the activation energy, the standard formation enthalpy, and the standard formation entropy. This indicates that its incorporation favors the degradation of steel; however, the results show a reduction in the corrosion rate. This effect is possible due to a synergistic effect by the formation of insoluble Fe-oxide layers that favor the formation of a Cr oxide layer at the Fe-oxide-metal interface, which limits the subsequent oxidation of Fe.

## 1. Introduction

Concentrated solar power (CSP) plants are one of the most promising solutions to use the potential of solar energy and convert it into electrical energy. In addition to their ability to generate electricity efficiently, their implementation has a positive environmental impact by reducing dependence on fossil fuels, significantly decreasing greenhouse gas emissions and other air pollutants. As a result, the carbon footprint associated with generating electrical energy through conventional methods is reduced, contributing to mitigating climate change and promoting a more sustainable future [1].

One of the main challenges of CSP plants is to ensure their continuous operation, which largely depends on their ability to store thermal energy. This storage is crucial, as it allows electricity production even when there is no solar radiation available, such as at night or on cloudy days. For this, CSP plants use thermal energy storage systems that capture heat by means of solar concentrators, using a special fluid called heat transfer fluid (HTF) [2,3]. HTFs in their flow path are in contact with metallic surfaces of various process components, such as storage tanks and pipes both for transport and within heat exchangers [4,5].

Since the beginning of the implementation of this technology, one of the main heat transfer fluids used is an oxyanionic mixture of nitrate salts. The nitrate salts used correspond to the NaNO_3_-KNO_3_ mixture, whose eutectic point (≈222 °C) is located at the equimolar ratio (46% NaNO_3_-54% KNO_3_, % by weight) [6,7]. However, around the eutectic point, the melting point of the mixture does not change significantly; for some reason, the 60% NaNO_3_-40% KNO_3_ (% by weight) mixture, known as Solar Salt, has been commonly used as HTF [4,8]. Because the oxyanionic mixture is likely to decompose into highly aggressive species for metallic materials (such as O_2_ and O^2−^), its operating range has been limited to temperatures below 600 °C [5,8,9,10,11,12]. Nevertheless, according to its equilibrium constant, the aggressive species are present, although in low concentrations, from the moment of the mixture melting. Furthermore, the increase in temperature increases its concentration and thus its aggressiveness towards the construction materials of CSP systems [3,12,13,14].

One of the main alloys used for the construction of pipelines used in CSP are Fe-based alloys due to their lower cost compared to Ni-based alloys, for example. However, the chemical stability of the surface of Fe-based alloys is dependent on the molten salt with which they are in contact. Various studies have shown that the corrosion mechanism in metal alloys, especially those based on iron, is related to the oxidation and dissolution processes of chromium (Cr), a key element for corrosion resistance in many alloys [15,16,17,18]. The same occurs in other molten salt systems [19,20,21,22,23], where the dissolution process causes the depletion of Cr on the surface of the alloy, leaving a Cr-depleted area with a spongy appearance due to the constant attempt to form a stable layer of chromium oxide. However, in the case of nitrate salts, because Fe oxides are insoluble, the formation of a layer of chromium oxide is possible, which allows reducing the corrosion rate of the alloy [24,25].

Improving the corrosion resistance of steels used in CSP plants is an active area of research. Some strategies to mitigate the effects of corrosion may include optimizing the composition of metal alloys by increasing the concentration of corrosion-resistant elements, using protective coatings, and developing salts with greater thermal stability and lower chemical aggressiveness. However, an important premise will always be the chemical stability of the materials when in contact with molten salts. For this reason, in this study, the kinetic aspects of the degradation process of ferritic steels immersed in Solar Salt at 400, 500, and 600 °C are analyzed using potentiodynamic polarization curves, and the thermodynamic aspects are determined using the Arrhenius equation and the expression of the Transition State Theory (expanded Arrhenius equation).

## 2. Materials and Methods

### 2.1. Materials Evaluated

The materials evaluated were the steels listed in Table 1. These consisted of ferritic steels (T2, T9, T11, T12, T22, T91) with different Cr and Mo contents. In addition, for comparison purposes, a medium carbon steel that did not contain Cr and Mo (A1) was included. The samples were obtained from seamless steel pipes specified in ASTM A213/A213M and ASTM A210/A210M [26,27]. The table reports the chemical composition of the main elements of interest for this study (Fe, Cr, and Mo), according to ASTM, in addition to the chemical composition determined by energy dispersive X-ray analysis (EDS) (JEOL model JSM IT 500).

### 2.2. Corrosive Medium

A 60% NaNO_3_-40% KNO_3_ (% by weight) mixture (Merck, Kenilworth, NJ, USA), known as Solar Salt, was used as a corrosive electrolyte. This is a binary mixture with one common anion. In terms of molar concentration, its composition is 64% NaNO_3_-36% KNO_3_ (% by weight) with a melting point of 238 °C. The tests were carried out at 400, 500, and 600 °C. This temperature range covers the typical operating temperatures of a concentrated solar power plant (≈400 °C) and the stability temperature of Solar Salt (≈600 °C). Therefore, the information obtained from this study will show the effect of increasing the operating temperature on the degradation process of the steels evaluated.

According to the phase diagram of the mixture, the eutectic point (222 °C) corresponds to the equimolar mixture (46% NaNO_3_-54% KNO_3_, molar %) [6,7]. However, since the solid-liquid boundary shows a wide range of low melting point mixtures, the 60% NaNO_3_-40% KNO_3_ (% by weight) composition was chosen for solar energy concentrator applications due to the lower cost of NaNO_3_ compared to KNO_3_ [8].

### 2.3. Potentiodynamic Polarization Curves

Potentiodynamic polarization curves were performed using a typical three-electrode arrangement. Samples of the different steels, with dimensions of 3 × 5 ×10 mm, were successively ground with abrasive paper from 120 to 600 grit and then washed with distilled water and acetone. To prepare the working electrodes, a Ni20Cr conducting wire was spot-welded to the metal samples. Platinum wire was used both as a counter electrode and a pseudo reference electrode. The conducting elements of the different electrodes were insulated with mullite tubes and sealed with ceramic cement. A vertical electric furnace was used for the tests, and the tests were performed under static air conditions. The tests were performed in triplicate.

Before the start of the tests, the electrode array was immersed in molten salt, contained in an alumina crucible, and allowed to stabilize for 15 min until reaching a stable potential. The working electrode was then polarized in the potential range from −300 to 1000 mV, with respect to its corrosion potential, at a scanning speed of 1 mV/s.

The electrochemical cell was coupled to a Gamry brand potentiostat-galvanostat, model Interface 1010 (Gamry Instruments, Warminster, PA, USA). The experiment was controlled with the Gamry Instrument Framework software and the electrochemical parameters (corrosion potential, Ecorr, corrosion current density, Icorr, anodic and cathodic slope, Ba and Bc) were obtained using the Tafel extrapolation procedure using the Gamry Echem Analyst software (Version 6.03).

**Figure 1:**
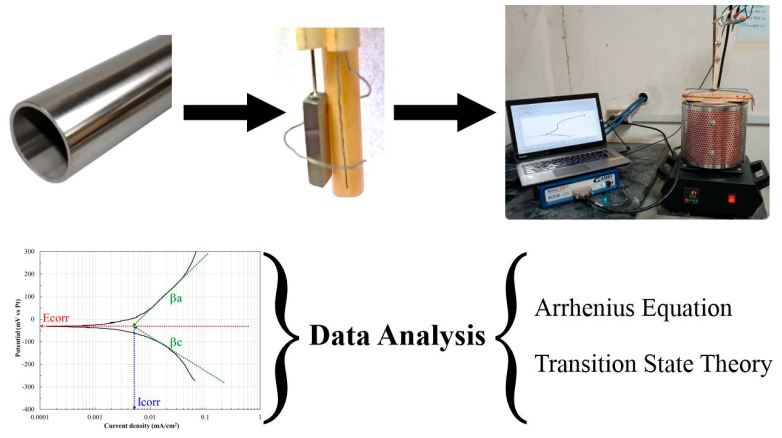
Schematic of the stages involved in the experimental procedure.

The dependence of the kinetic parameter (Icorr) on temperature was adjusted to the Arrhenius equation and the Transition State Theory in order to determine the thermodynamic parameters (Ea, ΔH°, and ΔS°) of the steel degradation process and its relationship with the chemical composition (Cr and Mo content). Figure 1 summarizes the stages involved in the experimental procedure described in the previous sections.

## 3. Results

### 3.1. Potentiodynamic Polarization

**Figure 2:**
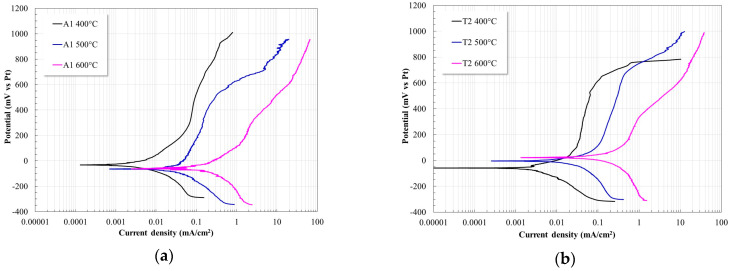

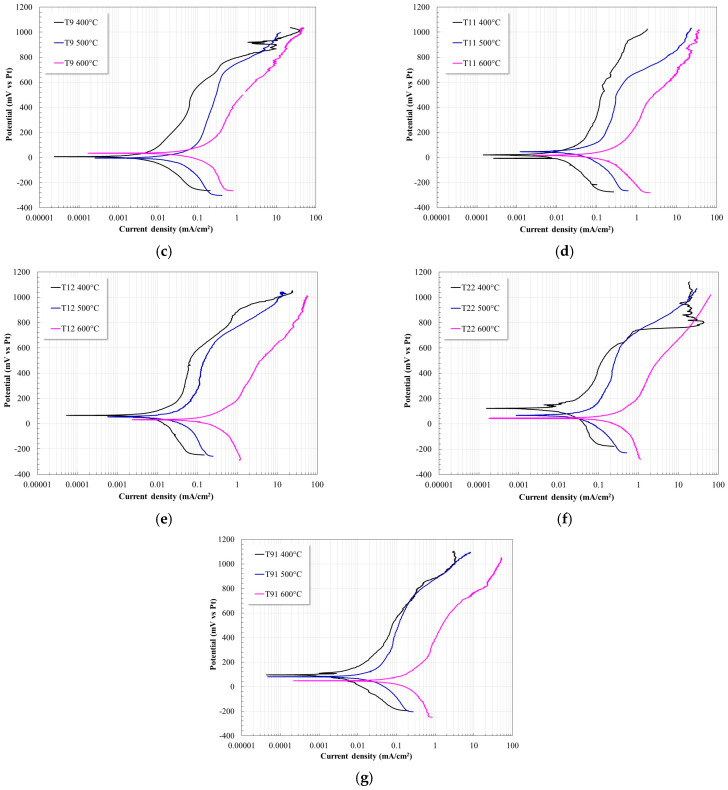
Potentiodynamic polarization curves of the steels evaluated in Solar Salt at 400, 500, and 600 °C.

Figure 2 shows the potentiodynamic polarization curves of the different steels evaluated in Solar Salt at 400, 500, and 600 °C. In general, it is observed that all of them show very similar behaviors both in their anodic and cathodic branches. In all cases, as the temperature increased, the polarization curves shifted to higher current densities, thereby suggesting an increase in the corrosion rate of the steels. Likewise, it is observed that above the corrosion potential, the steels showed an active behavior and at higher potentials the anodic branch tends to change its slope. This suggests that as polarization increases, the materials form more stable protective layers which modify the current–potential response.

**Figure 3:**
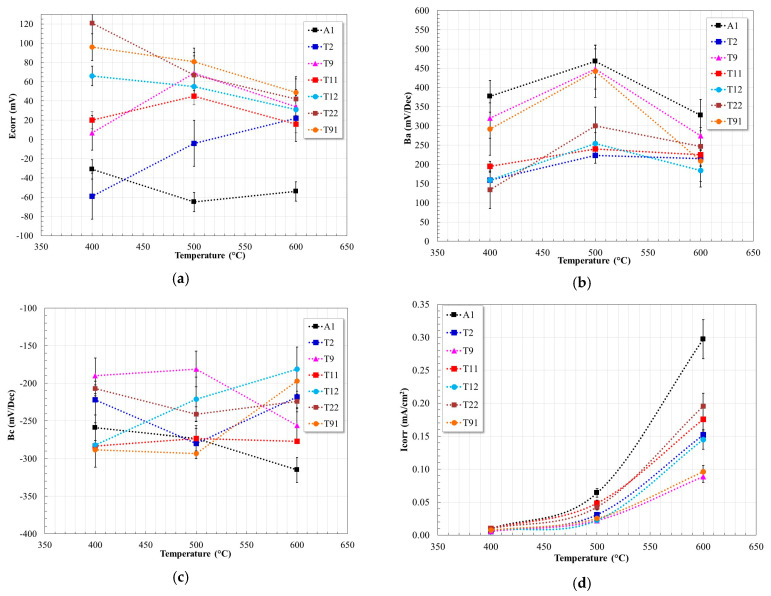
Electrochemical parameters, (**a**) Ecorr, (**b**) Ba, (**c**) Bc and (**d**) Icorr, obtained from the polarization curves of the steels evaluated in Solar Salt at 400, 500, and 600 °C.

Figure 3 shows the electrochemical parameters obtained from the polarization curves using the Tafel extrapolation method.

Regarding the corrosion potential (Figure 3a), it is observed that most of the steels (A1, T12, T22, T91) showed a shift to more active values with increasing temperature, others (T2) showed a shift to nobler values, and others an alternating behavior (T9, T11). It is common to expect a shift to more active values with increasing temperature due to greater electrochemical activity on the metal surface, as suggested by the shift of all polarization curves towards the region of higher current density. However, since Ecorr is the equilibrium potential obtained during a potential sweep, its value does not always coincide with the expected trend when measuring the potential under open circuit conditions (OCP); therefore, its interpretation is not necessarily the same.

In all cases, it is observed that the anodic slope (Figure 3b) tends to increase with increasing temperature (400 → 500 °C), which indicates an apparent reduction in the metal dissolution rate. However, at 600 °C the anodic slope decreases, which is indicative of an increase in the dissolution rate. The observed trend suggests that, at 500 °C, the amount of metal cations released is sufficient to oversaturate the surface; therefore, they precipitate as protective metal oxides. However, at 600 °C, the decrease in the anodic slope suggests an increase in the metal dissolution rate and a reduction in the protective capacity of the metal oxide layer. The trend of the anodic slopes is associated with the kinetics of the metal dissolution reactions, according to the reactions shown, and their interaction with other species in the melt (dissolved oxygen or oxide ion):

(1) $$Fe↔{Fe}^{2+}+2e^{-},$$

(2) $$Cr↔{Cr}^{2+}+2e^{-},$$

(3) $$Mo↔{Mo}^{6+}+6e^{-},$$

In general, the evolution of the cathodic slopes (Figure 3c) shows that their values are within a narrow range (≈−200 to ≈−300 mV/Dec). Because Solar Salt is an oxyanionic mixture, its aggressiveness depends on the equilibrium of the nitrate ion dissociation. It has been suggested that the nitrate ion dissociation may occur according to the following [9,10]:

(4) $${NO}_{3}^{-}↔{NO}_{2}^{+}+O^{2-},$$

(5) $${2NO}_{3}^{-}↔{2NO}_{2}+\frac{1}{2}O_{2}+O^{2-},$$

According to this proposal, the reducible species would be the nitrate ion and the nitronium ion. Therefore, during cathodic polarization, the presence of these species favors the following reduction reactions [9,10]:

(6) $${NO}_{3}^{-}+2e↔{NO}_{2}^{-}+O^{2-},$$

(7) $${NO}_{3}^{-}+e↔{NO}_{2}+O^{2-},$$

(8) $${NO}_{2}^{+}+e↔{NO}_{2},$$

However, the most widely used approach for the dissociation of nitrate salts is as follows [12,23,28]:

(9) $${NO}_{3}^{-}↔{NO}_{2}^{-}+\frac{1}{2}O_{2},$$

(10) $${2NO}_{2}^{-}↔{NO}_{2}+NO+O^{2-},$$

In this case, the reducible species would be the nitrate ion and the nitrite ion. Sustersic and coworkers [29] have suggested that the nitrite ion can be reduced according to one of the following reactions:

(11) $${NO}_{2}^{-}+e↔NO+O^{2-},$$

(12) $$2{NO}_{2}^{-}+4e↔N_{2}O+3O^{2-},$$

(13) $$2{NO}_{2}^{-}+6e↔N_{2}+4O^{2-},$$

(14) $$2{NO}_{2}^{-}+{NO}_{2}+e↔2NO+\frac{1}{2}O_{2}+O^{2-},$$

In the temperature range from 400 to 500 °C, the favorable reaction is the one involving the nitrate ion, and from 540 °C onwards, the reaction involving the nitrite ion [15]. However, in oxygen-rich atmospheres according to Le Chatelier’s Principle, the equilibrium of the decomposition reaction of the nitrate ion shifts to the left [12,30]. Because of this, the relevant reduction reaction under the conditions carried out in this work is the reduction in the nitrate ion.

According to the Lux-Flood acid/base model, if the concentration of the oxide ion (O^−2^) defines the basicity of the melt, then under the experimental conditions carried out in this work, the oxyanionic salts have a low basicity character which can increase with increasing temperature [12]. It has been reported that, under conditions evaluated here, the nitrite ion concentration is ≈3% by weight at 565 °C and ≈5.5% by weight at 600 °C [8,31].

The differentiation in the behavior of the steels resides mainly in the rate of the different processes that occur on the surface of each of them [29]. Where the rapidity of the anodic and cathodic reactions is reflected in the current density values obtained (Figure 3d). The observed trends show that below 600 °C, the Icorr values are low (<0.5 mA/cm^2^), and it is not possible to clearly define the effect of the alloy composition. However, at 600 °C, the Icorr values increase significantly, and it is possible to define three trends that are a function of the alloy composition. Namely, the steel without Cr and Mo content (A1) showed the highest corrosion rate, and those with a higher Cr content (≈9%) showed the lowest corrosion rate. In an intermediate region are those steels with a Cr content less than 2.25%. Since the Mo content in all alloyed steels ranged between 0.5 and 1.0%, it is not possible to define a significant effect. The trend of the Icorr values is in agreement with the indication of a low corrosivity of the nitrate salt at temperatures up to 500 °C, and confirms that, from 540 °C onwards, the aggressiveness of the melt is favored by the participation of the nitrite ion due to a shift in the nitrate-nitrite equilibrium, which increases the basicity of the melt [15,29,30].

Based on the *I_corr_* values, the corrosion rates (mm/year) were determined as suggested by ASTM G102 [32]:

(15) $$CR\;\left(\frac{mm}{year}\right)=K\;I_{corr}\frac{EW}{\rho }\;,$$

where, *K* = 3.27 × 10^−3^ (mm·g/μA·cm·year), *I_corr_* = corrosion current density (μA/cm^2^), *EW* = equivalent weight (dimensionless), and *ρ* = material density (g/cm^3^).

Equivalent weight was determined as follows:

(16) $$EW=\sum \frac{W_{i}}{n_{i}f_{i}},$$

where, *f_i_* = mass fraction of component *i* of the alloy, *W_i_* = atomic weight, and *n_i_* = valence.

**Figure 4:**
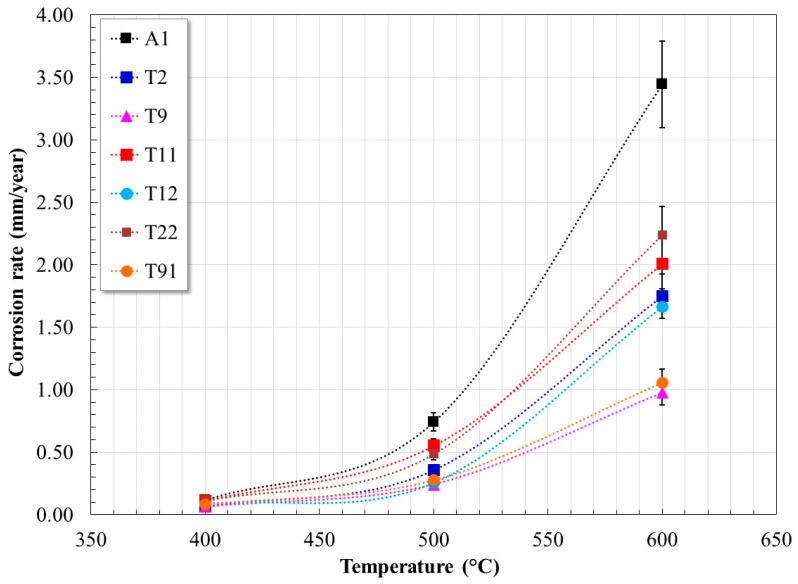
Effect of temperature on the corrosion rate of steels evaluated in Solar Salt at 400, 500, and 600 °C.

Figure 4 shows the corrosion rate values obtained. The corrosion rates expressed as a thinning rate show that at 400 °C, the thickness loss is around 0.01 mm/year, and at 500 °C, it reaches up to ≈0.7 mm/year. However, at 600 °C the thickness loss can vary between 1.0 and 3.5 mm/year depending on the alloy composition. It should be noted that there is no information in the literature indicating the acceptable limits of thickness loss for corrosion processes in molten salts. However, in the case of aqueous corrosion it has been suggested that a thickness loss between 0.5 and 1.0 mm/year is a normal degradation behavior of a material [33]. It is important to note that tests based on potentiodynamic polarization curves only reflect the initial behavior of the alloy, and this may vary with longer immersion times due to the possible formation of more stable corrosion product layers.

### 3.2. Thermodynamics of the Corrosion Process

Since temperature influences the kinetics of electrochemical processes, as well as the equilibrium of the reactions that take place during the degradation process, it is important to calculate the thermodynamic parameters that affect them. The use of the Arrhenius equation and the Transition State Theory allows the determination of the activation energy (*E_a_*), the standard transition enthalpy (Δ*H*°), and the standard transition entropy (ΔS°) [34,35]. The activation energy, *E_a_*, can be determined from the Arrhenius equation [35,36]:

(17) $$I_{corr}=A\exp\left(-\frac{E_{a}}{RT}\right),$$

where *E_a_* = activation energy of corrosion process (J/mol), *R* = universal gas constant, 8.31446 J/mol-K, *A* = frequency factor, *I_corr_* = corrosion current density, (A/cm^2^), and *T* = temperature, K.

**Figure 5:**
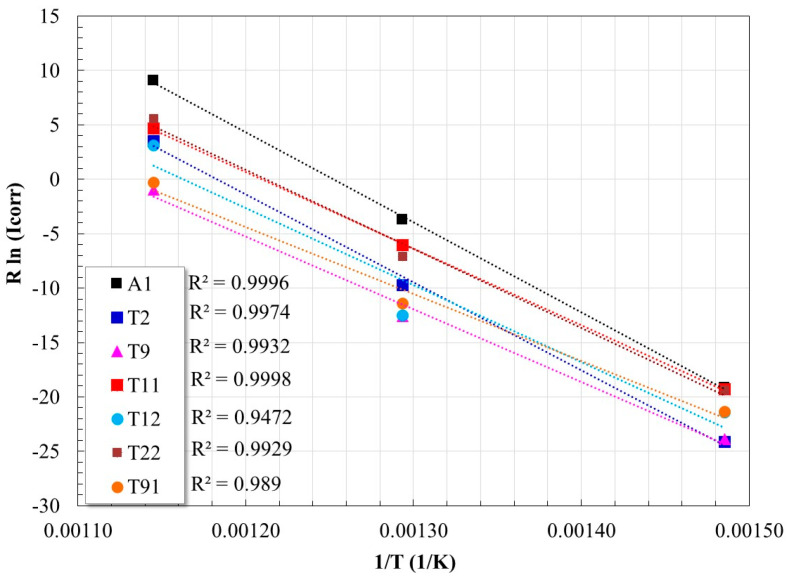
Arrhenius graph for calculating the activation energy (*E_a_*) of the corrosion process of the steels evaluated.

The activation energy is then determined from the slope of the graph of *R*ln(*I_corr_*) versus 1/*T* (Figure 5). Table 2 shows the values of *E_a_* calculated in this manner.

Since the Arrhenius kinetic model does not scientifically describe the frequency factor (*A*), the Transition State Theory has been used for its interpretation and calculation of the standard transition entropy (Δ*S*°), assuming that it remains constant within the temperature range under study [37,38]. Additionally, the standard transition enthalpy (Δ*H*°) is determined, whose value is very similar to that of the activation energy. In the case of corrosion, the Transition State Theory assumes the presence of a surface species in a transition state whose concentration is proportional to the concentration of a species present in the liquid phase [37]. The alternative form of the Arrhenius equation based on the Transition State Theory is as follows [36,39,40]:

(18) $$I_{corr}=\frac{RT}{Nh}\exp\left(\frac{∆S{}^{\circ}}{R}\right)\exp\left(-\frac{∆H{}^{\circ}}{RT}\right),$$

where, *h* = Planck constant (6.626176 × 10^−34^ J s) and *N* = Avogadro number (6.023 × 10^23^ mol^−1^).

A comparison with the Arrhenius equation shows that the standard enthalpy of transition (Δ*H*°) replaces the activation energy, and the frequency factor is equal to the following [38]:

(19) $$A=\frac{RT}{Nh}\exp\left(\frac{∆S{}^{\circ}}{R}\right),$$

**Figure 6:**
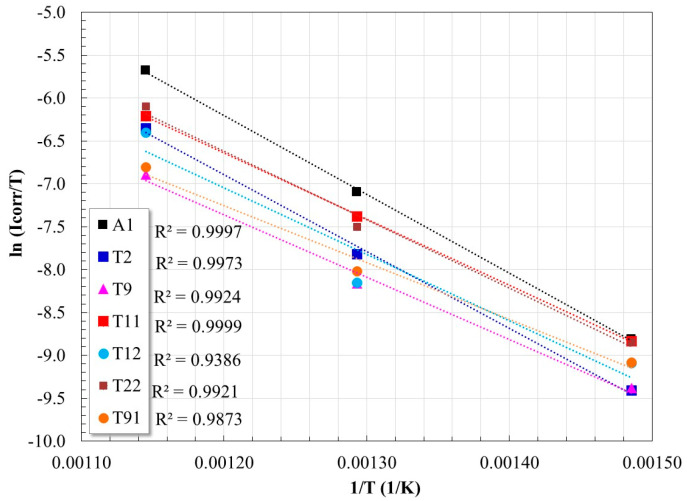
Graph for the calculation of Δ*H*° and Δ*S*° of the corrosion process of the steels evaluated.

By graphing ln (*I_corr_*/T) against 1/*T*, a straight line is obtained with a slope of (−∆*H*°/*R*), and an ordinate at the origin of [ln(*R*/*Nh*) + ∆*S*°/*R*] (Figure 6). Each line corresponds to the fit of the experimental data at the three temperatures evaluated in this study. From this procedure, the values of ∆*S*° and ∆*H*° reported in Table 2 were obtained.

**Figure 7:**
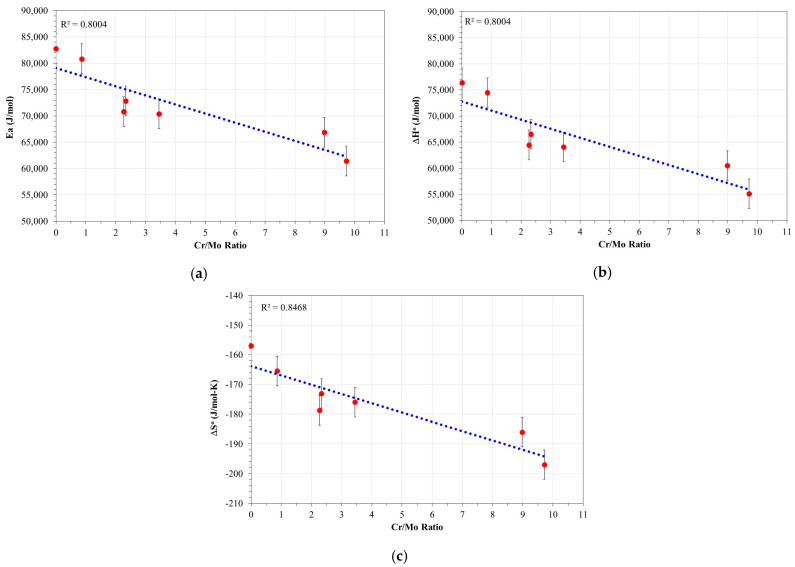
Variation of *E_a_*, Δ*H*°, and Δ*S*° as a function of the Cr/Mo ratio of the steels evaluated. (**a**) Variation of activation energy, (**b**) Variation of standard transition enthalpy, (**c**) Variation of standard transition entropy.

For a better understanding and interpretation of the influence of Cr and Mo content on the activation parameters, they were graphed against the Cr/Mo ratio of the alloy (Figure 7), with Cr/Mo ratios based on values obtained by EDS analysis (Table 1).

Activation energy (*E_a_*) represents the energy needed to trigger the rate-limiting step [40,41]. This parameter is crucial to understand the corrosion mechanisms, since an increase in *E_a_* indicates a higher energy barrier, which implies a higher energy requirement for the initiation of the corrosion process [42,43]. According to Table 2, typical values of *E_a_* for this type of alloy range between 60 and 100 kJ/mol, depending on the chemical composition, specifically the chromium (Cr) and molybdenum (Mo) content. In particular, the data presented in Figure 7a show that an increase in the Cr/Mo ratio correlates with a decrease in the *E_a_* values. This behavior suggests that the incorporation of Cr and Mo reduces the energy barrier, facilitating the initiation of the metal dissolution process. This effect could be attributed to the ability of these elements to modify the composition and structure of the passive layer formed on the surface of the alloy, promoting a more favorable environment for metallic dissolution.

**Figure 8:**
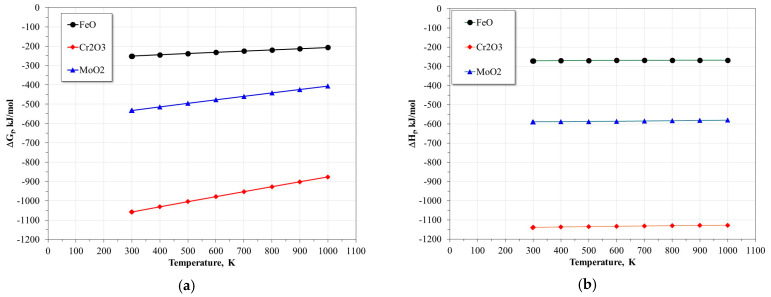
(**a**) Free energies of formation (ΔG_f_) and (**b**) enthalpies of formation (ΔH_f_) of the main metal oxides (FeO, Cr_2_O_3_, MoO_2_).

Assuming that at the beginning of the corrosion process the main metallic oxides formed are FeO, Cr_2_O_3_, and MoO_2_, their free energies of formation (Δ*G_f_*) and enthalpies of formation (Δ*H_f_*) as a function of temperature are shown in Figure 8 [44]. The Δ*G_f_* values suggest that, from a thermodynamic point of view, chromium (Cr) is the species with the greatest tendency to react to form its oxide (Cr_2_O_3_), followed by molybdenum (Mo), and iron (Fe). This is reflected in the more negative Δ*G_f_* values for Cr_2_O_3_, which indicate greater thermodynamic stability over a wide range of temperatures. In a complementary manner, the enthalpies of formation (Δ*H_f_*) confirm that the formation of Cr_2_O_3_ is a highly exothermic process, followed by MoO_2_ and FeO, suggesting that the incorporation of chromium and molybdenum in the alloy reduces the energy barrier for the onset of metallic dissolution, thus justifying the observed decrease in the activation energy (*E_a_*).

These findings are also consistent with previous studies indicating that Cr is soluble in oxyanionic salts present in these corrosive systems, while iron oxides are insoluble [15,30,45]. The rapid dissolution of Cr from the alloy surface leads to a local depletion of this element, which favors the formation of stable iron oxide layers. In turn, due to the low partial pressure of oxygen developed at the alloy-protective layer interface, the formation of a chromium oxide (Cr_2_O_3_) layer at the interface is possible. This oxide acts as a protective barrier that limits the further oxidation of iron (Fe) and stabilizes the alloy surface against the advance of the corrosive process. Similar behaviors have been observed in molten salt corrosion systems in other alloys, where the formation of mixed oxide layers, predominantly composed of Cr_2_O_3_ and insoluble iron oxides, plays a key role in corrosion resistance [19,20,21,22,23]. In this context, the decrease in the corrosion rate of steels can be directly attributed to the formation of this dual layer: a protective inner layer of chromium oxide at the alloy interface and an insoluble outer layer based on iron oxides.

**Figure 9:**
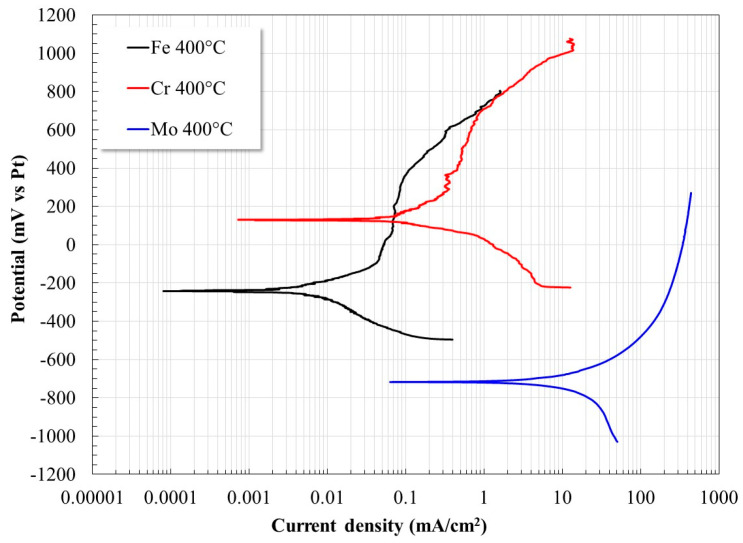
Polarization curves for pure Fe, Cr, and Mo at 400 °C.

Since the Mo concentration does not vary significantly, it is possible to infer an effect and behavior such as that observed with Cr. There are several studies that demonstrate the high reactivity of Mo in molten nitrate salts [8,46,47,48]. To corroborate the above, the same tests were performed with pure Fe, Cr, and Mo, and their polarization curves at 400 °C are shown in Figure 9. In general, for the case of Fe, it was possible to obtain its polarization curves at the three temperatures; however, in the case of Cr at 600 °C, it was not possible to perform the test due to its rapid dissolution, and in the case of Mo from 500 °C, the same was observed. In both cases, the dissolution was accompanied by a strong exothermic reaction and emanation of brown gases typical of the presence of nitrogen oxides. A comparison of the curves indicates that the reactivity of Cr is one order of magnitude greater than that of Fe, and in the case of Mo, its reactivity is three orders of magnitude greater. This supports the fact that Fe-based surface layers are more stable and limit the further dissolution of Cr and Mo. Furthermore, according to the oxidation reactions of the three metals, the number of electrons released by Mo is three times higher than that of Fe and Cr. This suggests an increase in the exchange current density and, therefore, the corrosion rate. The salts were analyzed after the tests at 600 °C, and it was found that in the case of Cr, the reaction products formed included Na and K chromates (K_3_Na(CrO_4_)_2_, Na_2_CrO_4_, NaCrO_2_, K_3_CrO_8_, K_2_Cr_2_O_7_, K_2_Cr_2_O_7_) and in the case of Mo, only the presence of Na_2_MoO_4_ was detected. This difference in the number of species formed may be correlated with the greater exothermic nature of the formation of Cr oxide that favored a greater increase in temperature and, therefore, in its reactivity.

On the other hand, the values of the standard transition enthalpy, Δ*H*°, obtained show a positive magnitude in all cases, indicating that the metallic dissolution is an endothermic process (Figure 7b) [34,42,43]. This behavior implies that the system requires an energy input to overcome the energy barrier associated with the formation of the activated complex or transition state. However, it is observed that the endothermic character of the process decreases with the increase in the chromium (Cr) and molybdenum (Mo) contents in the alloy. This suggests that the corrosion reaction requires less energy to occur, probably due to the influence of these elements on the structure and composition of the reactive surface. The reduction in the endothermic character may be related to the exothermic character of the formation of oxides such as Cr_2_O_3_ and MoO_2_, which are generated as secondary products of the metallic dissolution. This effect could explain the higher rate of formation of the activated complex in the presence of higher proportions of Cr and Mo.

Furthermore, it is notable that the magnitudes of the activation energy, Ea, and the standard transition enthalpy, Δ*H*°, are very similar (Figure 7a,b). According to Transition State Theory, this coincidence is expected, since ΔHo and Ea are usually closely related in chemical reactions where the only significant energy requirement is the breaking and formation of bonds in the transition state. This behavior has been reported by several authors, who argue that, for many chemical reactions, the activation energy and the standard transition enthalpy should be numerically equivalent under ideal conditions [49].

As for the values of standard transition entropy (ΔS°), a negative magnitude is observed in all cases, suggesting an increase in the order of the activated complex (Figure 7c) [42,43,50]. This behavior implies that the transition state is characterized by a higher degree of organization compared to the initial state of the reactant species. This phenomenon has been interpreted as an indication that the limiting step of the process is an association stage, where the molecules in the transition state present a more ordered and compact state due to specific interactions between the activated components [40,49,51,52].

The increase in the order of the system is directly correlated with the increase in the Cr/Mo ratio, indicating that chromium (Cr) plays a predominant role in the association and the increase in the order of the activated complex. This suggests that Cr favors the formation of a stable and ordered layer on the steel surface, which acts as a barrier limiting the direct access of the corrosive medium to the metal surface [49,53].

Although the incorporation of Cr and Mo reduces the energy level required for the corrosion process to take place, the results indicate a reduction in the corrosion rate. This may be because, at the beginning of the corrosion process, rapid dissolution of Cr and Mo occurs, thereby causing the surface layers of the steel to be free of these elements and, therefore, the Fe dissolution reaction is the main oxidation reaction that occurs. Subsequently, the Fe cations precipitate as insoluble oxides, thereby forming a protective layer on the surface [24].

This initial layer of insoluble Fe oxides limits the diffusion of the corrosive medium and reduces the partial pressure of oxygen at the interface. Under these conditions, the remaining Cr in the alloy diffuses towards the interface and reacts with the oxide ions (O^2−^), forming a layer of Cr_2_O_3_ at the interface between the Fe oxides and the base alloy. This chromium oxide acts as an additional barrier that inhibits both the diffusion and the subsequent oxidation of Fe [24,25].

At prolonged immersion times, iron and chromium oxides can react to form a FeCr_2_O_4_-rich composite layer, which has superior protective characteristics and reinforces the corrosion resistance of the alloy [8,54,55]. This behavior is consistent with previous reports, which suggest that Cr–Mo steel alloys used in applications such as solar concentrators should contain at least 9% by weight of Cr to ensure effective protection against corrosive environments [8].

These results highlight the importance of Cr and Mo content in optimizing the protective properties of steels in demanding environments, where the formation of stable and ordered layers plays a key role in mitigating corrosive effects.

## 4. Conclusions

Using potentiodynamic polarization curve tests, Arrhenius law, and the Transition State Theory, the kinetic and thermodynamic parameters of the degradation process of ferritic steels were obtained. The tests were carried out in Solar Salt at 400, 500, and 600 °C, and the results obtained showed the following:

The increase in temperature shifts the polarization curves towards higher current densities, indicating an increase in the corrosion rate of the steels.

The disturbance of the equilibrium in the anodic direction activates an active dissolution process and at higher disturbance potentials the anodic branch tends to change slope due to the development of more stable protective layers that modify the current–potential response.

The corrosion rates are low (< 0.5 mA/cm^2^) at temperatures below 600 °C and it is not possible to clearly define the effect of the alloy composition. However, at 600 °C, three trends are clearly defined. The highest corrosion rate was obtained in the absence of Cr and Mo, and when the Cr content was higher (≈ 9%) the lowest corrosion rate was obtained. In an intermediate position region are those steels with a Cr content of less than 2.25%.

The trend in the Icorr values is in accordance with the indication of low corrosivity of the nitrate salt at temperatures up to 500 °C and confirms that, at higher temperatures, the aggressiveness of the melt increases due to the participation of the nitrite ion, because of the displacement of the nitrate–nitrite equilibrium that increases the basicity of the melt.

The activation energy (*E_a_*) values indicate that the presence of Cr and Mo promotes the start of the metal dissolution process. This decrease in the energy barrier may be associated with the exothermic nature of the formation of Cr and Mo oxides.

According to the values of standard transition enthalpy (Δ*H*°), the metallic dissolution is an endothermic process and this decreases with the increase in Cr and Mo. This suggests that the corrosion reaction requires less energy and may be associated with the exothermic nature of the formation of the oxides.

The values of standard transition entropy, Δ*S*°, were negative, indicating an increase in the order of the activated complex. The order increased with the increase in the Cr/Mo ratio, suggesting the formation of a stable and ordered layer on the steel surface.

Cr and Mo reduce the energy level for the corrosion process to take place and, nevertheless, Cr reduces the corrosion rate. This contradictory effect is possible because the rapid dissolution of Cr and Mo causes the steel surface to be free of these elements, and the Fe dissolution reaction is the main oxidation reaction. Thus, the formation of insoluble oxides (Fe oxides) limits the diffusion of the melt and favors the formation of a Cr_2_O_3_ layer that prevents the subsequent diffusion and oxidation of Fe at the interface.

## Figures and Tables

**Table 1:** Cr and Mo content of the steels evaluated.

Material	Chemical Composition (% Weight) According to ASTM			Chemical Composition (% Weight) Determined by EDS		
	Fe	Cr	Mo	Fe	Cr	Mo
A1	Balance	0.00	0.00	Balance	0.00	0.00
T2	Balance	0.65	0.54	Balance	0.38	0.44
T9	Balance	9.00	1.00	Balance	8.98	0.00
T11	Balance	1.25	0.54	Balance	1.24	0.36
T12	Balance	1.02	0.54	Balance	1.11	0.49
T22	Balance	2.25	1.00	Balance	2.57	1.10
T91	Balance	8.75	0.95	Balance	9.23	0.95

**Table 2:** Thermodynamic parameters of the corrosion process of steels in Solar Salt.

Material	Cr	Mo	Cr/Mo	*E_a_*	Δ*H*°	Δ*S*°
	(% Weight)	(% Weight)		(J mol^−1^)	(J mol^−1^)	(J mol^−1^ K^−1^)
A1	0	0	0	82,739	76,400	−157
T2	0.38	0.44	0.86	80,828	74,489	−165
T9	8.98	0	8.98	66,868	60,529	−186
T11	1.24	0.36	3.44	70,393	64,054	−176
T12	1.11	0.49	2.26	70,799	64,460	−179
T22	2.57	1.1	2.34	72,845	66,507	−173
T91	9.23	0.95	9.72	61,445	55,107	−197

## Data Availability

The original contributions presented in this study are included in the article, further inquiries can be directed to the corresponding author.
